# The P2X7 Receptor: Central Hub of Brain Diseases

**DOI:** 10.3389/fnmol.2020.00124

**Published:** 2020-07-31

**Authors:** Roberta Andrejew, Ágatha Oliveira-Giacomelli, Deidiane Elisa Ribeiro, Talita Glaser, Vanessa Fernandes Arnaud-Sampaio, Claudiana Lameu, Henning Ulrich

**Affiliations:** Department of Biochemistry, Institute of Chemistry, University of São Paulo, São Paulo, Brazil

**Keywords:** P2X7 receptor, neurodegenerative diseases, psychiatric disorders, brain tumor, brain diseases, P2X7 receptor antagonists

## Abstract

The P2X7 receptor is a cation channel activated by high concentrations of adenosine triphosphate (ATP). Upon long-term activation, it complexes with membrane proteins forming a wide pore that leads to cell death and increased release of ATP into the extracellular milieu. The P2X7 receptor is widely expressed in the CNS, such as frontal cortex, hippocampus, amygdala and striatum, regions involved in neurodegenerative diseases and psychiatric disorders. Despite P2X7 receptor functions in glial cells have been extensively studied, the existence and roles of this receptor in neurons are still controversially discussed. Regardless, P2X7 receptors mediate several processes observed in neuropsychiatric disorders and brain tumors, such as activation of neuroinflammatory response, stimulation of glutamate release and neuroplasticity impairment. Moreover, P2X7 receptor gene polymorphisms have been associated to depression, and isoforms of P2X7 receptors are implicated in neuropsychiatric diseases. In view of that, the P2X7 receptor has been proposed to be a potential target for therapeutic intervention in brain diseases. This review discusses the molecular mechanisms underlying P2X7 receptor-mediated signaling in neurodegenerative diseases, psychiatric disorders, and brain tumors. In addition, it highlights the recent advances in the development of P2X7 receptor antagonists that are able of penetrating the central nervous system.

## Introduction

### The P2X7 Receptor

The investigation of receptors activated by adenosine triphosphate (ATP) has been largely widened since their discovery in 1960s by Geoffrey Burnstock. These receptors are classified into two main types: P1 and P2 receptors. P1 receptors are usually activated by adenosine, have seven transmembrane domains, and are coupled to G proteins. P2 receptors can be divided into two main subtypes, ionotropic P2X receptors and metabotropic P2Y receptors. P2X receptors subunits have just two transmembrane domains and are assembled as homo- or heterotrimers. Such as adenosine-activated P1 receptors, P2Y receptors are coupled to G proteins; however, their ligands are ATP/ADP/UTP/UDP-glucose (Knight, 2009).

Since the first cloning of the P2X7 receptor from a rat brain cDNA library (Surprenant et al., 1996), it is the most widely investigated purinergic receptor with the largest amount of specific pharmacological tools available (Sluyter and Stokes, 2011).

The *P2RX7* gene is comprised of 13 exons encoding the subunit with 595 amino acids in length that in humans is located at chromosome position 12q24.31 and in mice at chromosome 5. The human *P2RX7* gene is located at the chromosome position also associated with inflammatory and psychiatric disorders (Barden et al., 2006; Lucae et al., 2006). Each one of the three subunits has intracellular amino and carboxyl termini with two hydrophobic transmembrane domains, with a long glycosylated extracellular loop between them, comprising the ATP-binding site. In addition, the P2X7 receptor usually assembles as homotrimer (Sluyter and Stokes, 2011). However, it can also form heteromeric interactions with P2X4 receptor subunits as evidenced in 2007 by Guo et al. (2007) and later confirmed by Schneider et al. (2017).

P2X7 receptor activity is triggered by high concentrations (ranging around 0.05–1 mM) of extracellular adenosine 5′-triphosphate (ATP), mediating the rapid influx of Na^+^ and Ca^2+^ and efflux of K^+^, and other cations (Burnstock and Kennedy, 2011). Upon long activation, the P2X7 receptor can open pores large enough to allow the passage of organic ions like N-methyl-D-glucamine (NMDG^+^), choline^+^ and fluorescent dyes such as ethidium^+^ and YO-PRO-12^+^ (Alves et al., 2014).

Available tools for P2X7 receptor research lack specific agonists. Due to this problem, many literature data need to be carefully analyzed. Studies regarding the activation of P2X7 receptors use agonists, such as ATP and 2′(3′)-O-(4-Benzoylbenzoyl)adenosine 5′-triphosphate (Bz-ATP). ATP is a broad agonist for P2X receptors. Bz-ATP is 10–50 times more potent than ATP in activating P2X7 receptors. Besides activating P2X7 receptors, this compound acts as an agonist for P2Y11, P2X1, 2 and 4, and as a weak agonist for P2X5 receptors. Additionally, EC_50_ values for both agonists vary between species. Bz-ATP, for example, activates rat and human P2X7 receptor at 10 times greater concentration than mice P2X7 receptor (Burnstock and Verkhratsky, 2012). As indicated in Table 1, some P2X7 receptor antagonists also lack specificity. The widely used Brilliant Blue G (BBG) also antagonizes P2X1, P2X2, P2X3, and P2X4 receptors besides the P2X7 receptor. However, the IC_50_ for the P2X7 receptor is 8–50 times lower compared with other receptors. A-740003, A-438079 and A-804598 are selective for the P2X7 receptor (Burnstock and Verkhratsky, 2012).

**TABLE 1:** P2X7 receptor antagonists.

Structure	Compound/IUPAC name	BBB-penetrant	Type	References
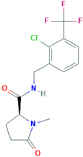	**GSK-1482160** (2S)-N-[[2-chloro-3-(trifluoromethyl)phenyl]methyl]-1-methyl-5-oxopyrrolidine-2-carboxamide	Yes	Preferential P2X7 receptor antagonist	Territo et al., 2017; Kim et al., 2019

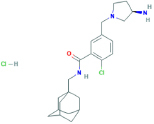	**GSK-314181A** N-(1-adamantylmethyl)-5-[[(3R)-3-aminopyrrolidin-1-yl]methyl]-2-chlorobenzamide;hydrochloride	Yes	Preferential P2X7 receptor antagonist	Broom et al., 2008; Kim et al., 2019

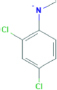	**Compound 16 (GSK)** (2,4-dichlorophenyl)-methylazanide	Yes	Preferential P2X7 receptor antagonist	Beswick et al., 2010; Kim et al., 2019

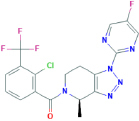	**JNJ-54175446** [2-chloro-3-(trifluoromethyl)phenyl]-[(4R)-1-(5-fluoropyrimidin-2-yl)-4-methyl-6,7-dihydro-4H-triazolo[4,5-c]pyridin-5-yl]methanone	Yes	Preferential P2X7 receptor antagonist	Letavic et al., 2017; Kim et al., 2019

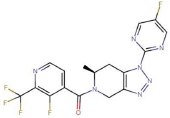	**JNJ-55308942** (S)-(3-fluoro-2-(trifluoromethyl)pyridin-4-yl)(1-(5-fluoropyrimidin-2-yl)-6-methyl-1,4,6,7-tetrahydro-5H-[1,2,3]triazolo[4,5-c]pyridin-5-yl)methanone	Yes	Non-selective P2X7 receptor antagonist (also binds to P2X1, P2X2, P2X3, P2X2/3, and P2X4 receptors)	Chrovian et al., 2018

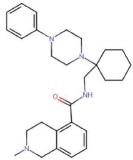	**JNJ-42253432** 2-methyl-N-([1-(4-phenylpiperazin-1-yl)cyclohexyl]methyl)-1,2,3,4-tetrahydroisoquinoline-5-carboxamide	Yes	Preferential P2X7 receptor antagonist	Letavic et al., 2013; Lord et al., 2014

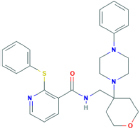	**JNJ-47965567** N-[[4-(4-phenylpiperazin-1-yl)oxan-4-yl]methyl]-2-phenylsulfanylpyridine-3-carboxamide	Yes	Preferential P2X7 receptor antagonist	Bhattacharya et al., 2013; Letavic et al., 2013; Kim et al., 2019

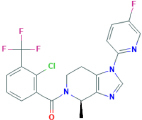	**JNJ-54166060** [2-chloro-3-(trifluoromethyl)phenyl]-[(4R)-1-(5-fluoropyridin-2-yl)-4-methyl-6,7-dihydro-4H-imidazo[4,5-c]pyridin-5-yl]methanone	Yes	Preferential P2X7 receptor antagonist	Swanson et al., 2016; Kim et al., 2019

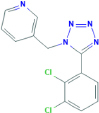	**A-438079** 3-[[5-(2,3-dichlorophenyl)tetrazol-1-yl]methyl]pyridine	Yes	Preferential P2X7 receptor antagonist	Nelson et al., 2006; Kim et al., 2019

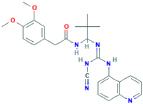	**A-740003** N-[1-[(Z)-[(cyanoamino)-(quinolin-5-ylamino)methylidene]amino]-2,2-dimethylpropyl]-2-(3,4-dimethoxyphenyl)acetamide	Yes	Preferential P2X7 receptor antagonist	Honore et al., 2006; Kim et al., 2019

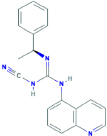	**A-804598** 1-cyano-2-[(1S)-1-phenylethyl]-3-quinolin-5-ylguanidine	Yes	Preferential P2X7 receptor antagonist	Donnelly-Roberts et al., 2009; Able et al., 2011; Kim et al., 2019

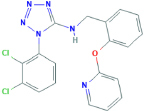	**A-839977** 1-(2,3-dichlorophenyl)-N-[(2-pyridin-2-yloxyphenyl)methyl]tetrazol-5-amine	Yes	Preferential P2X7 receptor antagonist	Honore et al., 2009; Kim et al., 2019

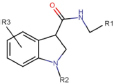	**AFC-5128** indol-3-carboxamide derivative, chemical nomenclature disclosed	Yes	Preferential P2X7 receptor antagonist	Fischer et al., 2016

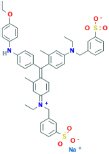	**Brilliant blue G (BBG)** 3-[[4-[(E)-[4-(4-ethoxyanilino)phenyl]-[4-[ethyl-[(3-sulfonatophenyl)methyl]azaniumylidene]-2-methylcyclohexa-2,5-dien-1-ylidene]methyl]-N-ethyl-3-methylanilino]methyl]benzenesulfonate	Yes	Non-selective P2X7 receptor antagonist (also binds to P2X1, P2X2, P2X3 and P2X4 receptors)	Savio et al., 2018; Kim et al., 2019

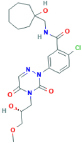	**CE-224, 535** 2-chloro-N-[(1-hydroxycycloheptyl)methyl]-5-[4-[(2R)-2-hydroxy-3-methoxypropyl]-3,5-dioxo-1,2,4-triazin-2-yl]benzamide	No	High selective P2X7 receptor antagonist (500-fold over P2X1 and P2Y1 receptors)	Savall et al., 2015; Kim et al., 2019

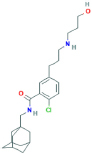	**AZD9056** N-(1-adamantylmethyl)-2-chloro-5-[3-(3-hydroxypropylamino)propyl]benzamide	No	Preferential P2X7 receptor antagonist	Bhattacharya, 2018; Kim et al., 2019

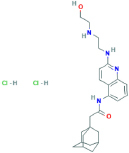	**AZ-10606120** 2-(1-adamantyl)-N-[2-[2-(2-hydroxyethylamino)ethylamino]quinolin-5-yl]acetamide;dihydrochloride	Not found	Negative allosteric modulator of the human P2X7 receptor.	Kim et al., 2019

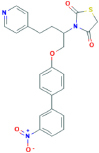	**AZ-11645373** 3-[1-[4-(3-nitrophenyl)phenoxy]-4-pyridin-4-ylbutan-2-yl]-1,3-thiazolidine-2,4-dione	Not found	Preferential P2X7 receptor antagonist (500 times less effective in rat than in human P2X7 receptors)	Kim et al., 2019

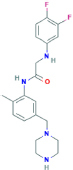	**GW791343** 2-(3,4-difluoroanilino)-N-[2-methyl-5-(piperazin-1-ylmethyl)phenyl]acetamide	Not found	Negative activity modulator of human P2X7 receptors, positive activity modulator of rat P2X7 receptors	Kim et al., 2019

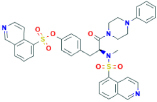	**KN-62** [4-[(2S)-2-[isoquinolin-5-ylsulfonyl(methyl)amino]-3-oxo-3-(4-phenylpiperazin-1-yl)propyl]phenyl] isoquinoline-5-sulfonate	Not found	Preferential human P2X7 receptor antagonist, however with low affinity to rat P2X7 receptors	Kim et al., 2019

*BBB: blood brain barrier; IUPAC: International Union of Pure and Applied Chemistry.*

Another limitation regarding P2X7 receptors studies is antibody specificity. Available antibodies against the P2X7 receptor are polyclonal, which are prone to cross-reactivity, or monoclonal. Although they did not detect P2X7 receptors in knockout (KO) mice, these monoclonal antibodies failed to consistently and reliably detect and/or block P2X7 receptor signaling pathway in WT mice (Sim et al., 2004; Li et al., 2020). There are at least two P2X7 receptor KO mice commercially available. In the GlaxoSmithKline strain, generated by Chessell et al. (2005), a lacZ transgene and neomycin cassette were inserted into exon 1 (Chessell et al., 2005). These animals express the P2X7K receptor isoform and lack the P2X7A receptor isoform. P2X7K is widely expressed by T lymphocytes, and GlaxoSmithKline P2X7 receptor KO mice possess enhanced P2X7 receptor-mediated responses in T cells. The other available strain from Pfizer, generated by Solle et al. (2001) by inserting a neomycin cassette into exon 13, lacks both P2X7A and K receptor isoforms; however these animals express P2X7 13B and 13C isoforms in the brain and other tissues (Solle et al., 2001; Bartlett et al., 2014). The P2X7 13B isoform was reported to negatively affect P2X7A receptor activity (Masin et al., 2012). Therefore, P2X7 receptor KO mice should be used carefully as a tool to assess P2X7 receptor involvement in brain and inflammation.

Nowadays, P2X7 receptor expression is known to be broadly present throughout diverse tissues and cells, including CNS, such as microglia, oligodendrocytes, Schwann cells, and possibly in astrocytes and neurons. The latter one is still controversial discussed, and various works are still trying to clarify the issue (see Sluyter and Stokes, 2011). Despite several works that demonstrate the presence of P2X7 receptor in neurons (Deuchars et al., 2001; Sperlágh et al., 2002; Wirkner et al., 2005; Yu et al., 2008), its expression and functionality are widely debated (Sim et al., 2004; Anderson and Nedergaard, 2006; Illes et al., 2017; Metzger et al., 2017b). This outlook becomes strengthened when immunoreactivity for this receptor in P2X7 receptor KO strains was detected, evidencing low specificity of anti-P2X7 receptor antibodies (Anderson and Nedergaard, 2006). Recent works with improved methodologies did not find any expression of P2X7 receptors in neurons (Rubini et al., 2014; Kaczmarek-Hajek et al., 2018; Khan et al., 2018). Similarly, the presence of functional P2X7 receptors in astrocytes is also debated c). It is well known, however, that oligodendrocytes and microglia express functional P2X7 receptors (Lord et al., 2015; He et al., 2017; Metzger et al., 2017a; Kaczmarek-Hajek et al., 2018).

### Variants of the P2X7 Receptor

**FIGURE 1:**
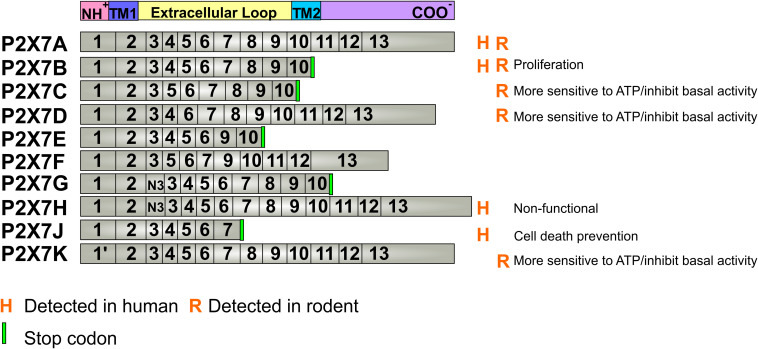
P2X7 receptor splicing variants. The P2X7 receptor has 10 different isoforms derived from alternative splicing and mutations of the 13 exons of the gene. The P2X7A isoform is the native form, expressed in every mammal species. The detected alterative isoforms in humans are P2X7B, H and J, while in rodents, these are P2X7B, C, D and K. The mutations that lead to a stop codon insertion, originate a shortened P2X7 receptor at the carboxy-terminal domain and cannot form pores that induce cell death. P2X7G and H present a copy of exon 3 (N3) near the amino-terminal. Known basic functions for each isoform are described at the right site of the panel. Aminoterminal (NH^+^), Carboxyterminal (COO^–^), Transmembrane passage 1 (TM1), Transmembrane passage 2 (TM2).

The P2X7 receptor has 10 different alternative splicing isoforms named from P2X7A to P2X7K, the latter has only been identified in rodents (Figure 1). The full-length isoform is the P2X7A one. In humans, P2X7B, P2X7H, and P2X7J are the only subunits reported as expressed proteins (Feng et al., 2006; Adinolfi et al., 2010) (Figure 1).

The P2X7B isoform is a truncated form, when compared with P2X7A (Cheewatrakoolpong et al., 2005), and assemble as functional channels that cannot form large pores as P2X7A, playing roles in cell proliferation (Adinolfi et al., 2010). The P2X7H is nonfunctional ion channels (Cheewatrakoolpong et al., 2005), whereas the P2X7J can assemble with other splicing variants forming non-functional heterotrimeric receptors (Feng et al., 2006) that are involved in protection against ATP-induced cell death (Feng et al., 2006; Guzman-Aranguez et al., 2017).

In mice, four splice variants were detected (P2X7B, P2X7C, P2X7D, and P2X7K), besides the canonical P2X7A one. Most of the modifications between isoforms are comprised within the extracellular loop domain. P2X7D and P2X7B can assemble to P2X7A and negatively affect the basal activity of the P2X7 receptor. However, if not assembled to P2X7A, they assemble as receptors forms that show both increased activity and higher sensitivity to agonists (Schwarz et al., 2012; Xu et al., 2012), like the rat P2X7K variant (Nicke et al., 2009). Restricted P2X7 receptor variants present multiple mutations, such as the P2X7 receptor-2 variant that contains H270R and A348T mutations, and the P2X7 receptor-4 variant that has H155Y, H270R, A348T, and Q460R mutations (Stokes et al., 2010). These variants in heterologous expression cells also exhibited larger agonist-induced ion currents and dye uptake with a similar agonist sensitivity (Jiang et al., 2013).

**FIGURE 2:**
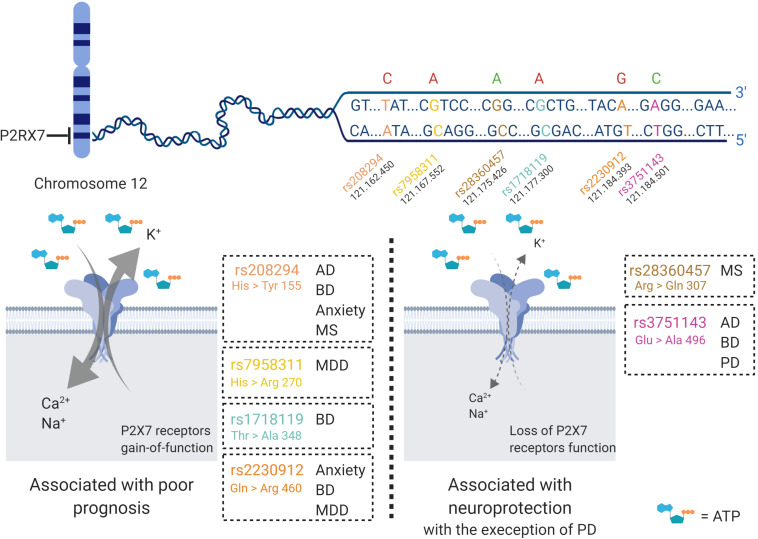
P2X7 receptor single nucleotide polymorphisms (SNP) in brain diseases. Various P2X7 receptor SNPs have been detected and studied in humans. The gene that encodes the P2X7 receptor is located at chromosome 12, and at least seven of the SNPs are related to neurological disorders, such as Alzheimer’s disease (AD), bipolar disorder (BD), anxiety, multiple sclerosis (MS), major depressive disorder (MDD) and Parkinson’s disease (PD). The red letters represent SNPs that potentialize the response of the P2X7 receptor upon binding to its ligand and generate increased cell death and worsening of diseases. Further, green letters are in line with decreased Ca^2+^ inflow due to loss of function of the P2X7 receptor, and usually lead to neuroprotection. The SNPs are named rs208294, rs7958311, rs1718119, rs2230912, rs28360457, and rs3751143. Created with BioRender.com.

Some alternative splicing isoforms of P2X7 receptor show diverse downstream signaling properties. Moreover, P2X7 receptor function varies among human individuals because there are some polymorphisms that can result in loss- or gain-of-function (Figure 2). Single nucleotide polymorphisms (SNPs) are widespread in the human P2X7 receptor; some of them are non-synonymous, meaning that there is a change in the amino acid sequence, generating a point mutation. Some of those mutations are related to altered susceptibility to various diseases, shedding new light on the underlying disease mechanisms (Jiang et al., 2013). In this article, we review SNPs involved in Alzheimer’s disease (AD) (rs208294, rs3751143), Parkinson’s disease (PD) (rs3751143), multiple sclerosis (MS) (rs208294, rs28360457), depressive disorder (rs7958311, rs2230912), anxiety (rs208294, rs2230912), and bipolar disorder (BD) (rs208294, rs1718119, rs2230912, rs3751143) (Figure 2).

### P2X7 Receptor Function

P2X7 receptor activation induces a number of well-established downstream signaling events in various human cell types. The opening of the channel leads to an increase in the concentration of cytosolic Ca^2+^ ([Ca^2+^]_i_), responsible for a number of P2X7 receptor-induced responses, as AKT activation in astrocytes (Jacques-Silva et al., 2004). Phospholipase (PL) C and A2 (Andrei et al., 2004), src kinase, p38, acid sphingomyelinase (Bianco et al., 2009), caspase 1 (Keller et al., 2008), and gasdermin (Evavold et al., 2018) are involved in P2X7 receptor intracellular signaling.

P2X7 receptor activity mediates cell proliferation and death, rapid and reversible phosphatidylserine exposure, membrane blebbing, release of microparticles and exosomes and multinucleated cell formation, as well as the formation of reactive oxygen and nitrogen species (Sluyter and Stokes, 2011).

### P2X7 Receptor in Neuroinflammation

The P2X7 receptor is highly expressed in microglial cells (Lord et al., 2015; He et al., 2017). In healthy tissues, the concentration of extracellular ATP is low at the nanomolar range (Falzoni et al., 2013). Conversely, under stress and cellular damage, the ATP concentration increases considerably, resulting in P2X7 receptor activation. Therefore, it is hypothesized that P2X7 receptor acts as a silent receptor once its activation only occurs in pathological states when there is a rise of extracellular ATP concentrations (Bhattacharya and Biber, 2016).

In high concentrations, extracellular ATP can act as a damage-associated molecular patterns (DAMPs) and activate P2X7 receptor (Falzoni et al., 2013). DAMP signal activates the transcription factor NF-κB in the nucleus, which consequently promote the upregulation of proinflammatory cytokines, pro-IL-1β and pro-IL-18, and NLRP3 protein (Jo et al., 2016). Although the precise mechanism is not completely understood, P2X7 receptor it is one of the most potent activators of the NRLP3-associated inflammasome (He et al., 2017). P2X7 receptor activation induces K^+^ efflux, which is needed for efficient NLRP3 inflammasome activation (Gustin et al., 2015). NLRP3 inflammasome trigger the activation of caspase-1, which causes the maturation of IL-1β and IL-18 and, consequently, increasing proinflammatory cytokine release (Bernier, 2012; Jo et al., 2016; He et al., 2017; Bhattacharya et al., 2018). This signaling appears to be in functional in microglia and not astrocytes (Gustin et al., 2015). Additionally, the P2X7 receptor may also stimulate the release of TNF, IL-6, CCL2, CCL3, and CXCL2 (Suzuki et al., 2004; Kataoka et al., 2009; Shiratori et al., 2010; Shieh et al., 2014).

It is clear that the P2X7 receptor can modulate the neuroinflammation induced by LPS, once P2X7 receptor blockade may reduce inflammatory mediators release (Bianco et al., 2006; Choi et al., 2007; Monif et al., 2009; He et al., 2017; Yang et al., 2018). Some works showed that LPS enhanced P2X7 receptor expression (Choi et al., 2007; Yang et al., 2018), whereas other studies reported downregulation of P2X7 receptor expression (Bianco et al., 2006; He et al., 2017). Similarly to the LPS-induced effects, P2X7 receptor overexpression was sufficient to trigger microglial activation in primary microglia derived from hippocampus (Monif et al., 2009). Interestingly, a recent study evidenced that the selective P2X7 receptor antagonist, JNJ-55308942, inhibited neuroinflammation development induced in different rodent models by LPS, BCG or chronic stress (Bhattacharya et al., 2018). Recently, efforts were made to detect *in vivo* neuroinflammation. Therefore, radioligands targeting P2X7 receptor were used as a tool to identify brain areas undergoing inflammatory processes. [^18^F]-JNJ-64413739 and ^11^C-GSK1482160 were promising in detecting areas of neuroinflammation upon LPS-stimulation of in rodents (Territo et al., 2017; Berdyyeva et al., 2019).

One of the possible pathways for ATP release is from dying cells. Interestingly, diseases that present degeneration of neural cells, as neurodegenerative diseases, psychiatric disorders, and brain tumors, as presented below, may present high local concentrations of extracellular ATP and stimulate pathophysiological P2X7 receptor activity. In view of that, here, we provide evidence that AD, PD, MS, depression, and brain tumors present increased P2X7 receptor expression. P2X7 receptor signal amplification in these diseases is proposed.

## P2X7 Receptor Roles in Neurodegenerative Diseases

Purinergic receptors play a significant role in neurodegenerative diseases (Oliveira-Giacomelli et al., 2018). P2X7 receptors participate in neurodegenerative, neuroinflammatory and neurogenic processes, tightly related to disease development and repair.

### Alzheimer’s Disease

Alzheimer’s disease is the most common form of dementia in the elderly population (Ballard et al., 2011; Beinart et al., 2012), representing a serious public health problem. Recent estimative indicates that approximately 50 million people have AD worldwide, and this number is expected to reach 132 million by 2050 (Alzheimer’s Association, 2015). Processes that trigger AD may start decades before the onset of initial symptoms of dementia (Goedert and Spillantini, 2006; De Felice, 2013), reinforcing the importance of sensitive diagnostic tools for more effective therapeutic interventions.

The main clinical symptom in AD is the cognitive decline, which begins with recent memory lapses, and proceeds with progressively intensified memory loss to total physical dependence. Familial AD (∼5% of all cases) is more severe and initiates earlier than the sporadic form, affecting people from 40 years of age on. Most patients are sporadic cases, presenting AD symptoms from 65 years of life on, and aging is indicated as the leading risk factor for the disease (Evans et al., 1989). The pathophysiologic generation of the neurotoxic β-amyloid oligomers (AβO) by sequential amyloid precursor protein (APP) proteolysis is involved in the development of AD. Familial AD has been directly related to mutations in the genes of APP and presenilin 1 and 2 (Levy-Lahad et al., 1995; Sherrington et al., 1995). The etiology of AD is an association between genetic and environmental factors (Selkoe, 2004; Roberson and Mucke, 2006) which turns disease treatment more difficult. Indeed, the drugs currently available to treat AD have only palliative effects and consist of acetylcholinesterase inhibition to optimize cholinergic activity (Knapp et al., 1994; Rogers and Friedhoff, 1996; Trinh et al., 2003), and the NMDA receptor antagonist memantine (Cosman et al., 2007; Lipton, 2007; Parsons et al., 2007; Xia et al., 2010). Therefore, the development of more effective drugs for AD treatment is needed.

There is evidence that inflammation plays a vital role in AD (Lucin and Wyss-Coray, 2009), as well as in the modulation of neurogenesis (Mishra et al., 2015). Interestingly, there is a significant influence of microglia in both processes (Nunan et al., 2014; De Lucia et al., 2016). AβO also activates microglia, resulting in secretion of pro-inflammatory cytokines, such as tumor necrosis factor alpha (TNF-α) and IL-1β (Ledo et al., 2013, 2016). Microglial activation may not only compromise their clearance ability (Heneka et al., 2010) but also, surprisingly, contribute to the propagation of AβO in the cerebral parenchyma (Joshi et al., 2014). Interestingly, the P2X7 receptor is involved in these features and in AD as discussed in the following.

**FIGURE 3:**
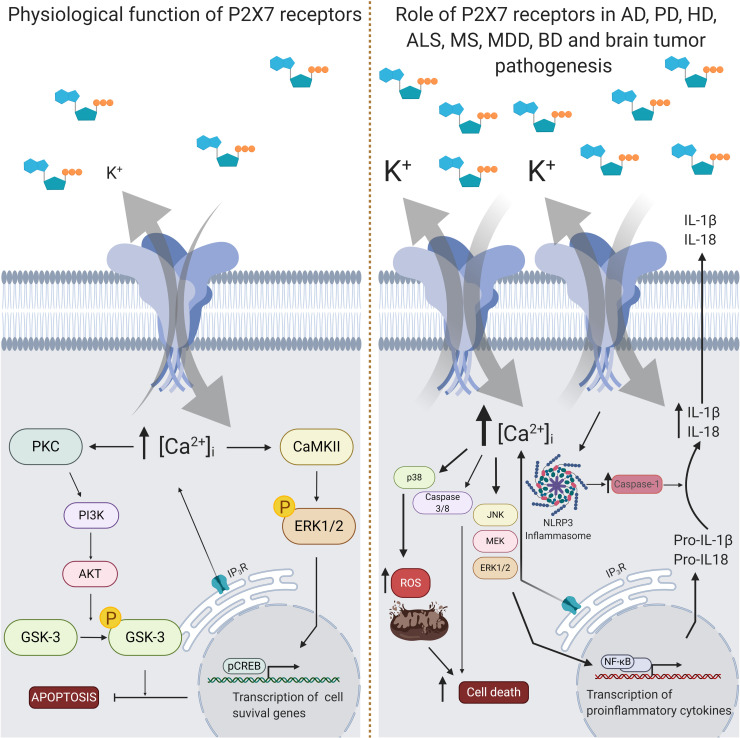
Intracellular signaling pathways triggered by P2X7 receptor activity. The P2X7 receptor is assembled as a homotrimeric protein, and upon ATP binding, receptor subunits change their conformational state and open a pore for the entrance of cations, mainly Ca^2+^. In physiological conditions (left panel), the increase of intracellular Ca^2+^ concentration levels ([Ca^2+^]_i_) leads to the activation of some kinases, like protein kinase C (PKC) and calcium-calmodulin kinase II (CaMKII), which phosphorylates and activates phosphoinositide 3-kinase (PI3K), extracellular signal-regulated kinases 1/2 (ERK1/2), protein kinase B (AKT) and glycogen synthase kinase 3 (GSK3). This signal transduction results in inhibition of apoptosis or increase of the transcription of cell survival related genes. In pathological conditions (right panel), such as in Alzheimer’s disease (AD), multiple sclerosis (MS), major depressive disorder (MDD) and Parkinson’s disease (PD), P2X7 receptor expression rates are increased. Activation of the P2X7 receptor in AD animal model results in increased release of interleukin 1β (IL-1β) and reactive oxygen species (ROS), and augmented inhibition of GSK3. IL-1β release depends on the formation of the NLRP3 inflammasome together with the activation of the nuclear factor kappa-light-chain-enhancer activated B cells (NF-κB). In ALS, P2X7 receptor activation also induces overproduction of ROS and ERK1/2 signaling. Administration of P2X7 receptor antagonists has been suggested to benefit specific features of AD, PD, MS, MDD, and BD, like improvement of behavior and neuroinflammation. Nevertheless, high concentrations of P2X7 receptor agonists may also enhance *in vitro* cytotoxic effects of temozolomide, a drug of choice for glioblastoma treatment. Created with BioRender.com.

Increased P2X7 receptor expression and activation have been involved in the progression of several neurodegenerative diseases, including AD (Savio et al., 2018). Accordingly, P2X7 receptor expression is increased in the brain of AD patients and appears to be concentrated in areas of higher density of amyloid plaques, co-localized with activated microglia (McLarnon et al., 2006). P2X7 receptors expression are also upregulated in the hippocampus of two animal models of AD, such as transgenic mice that express the human APP bearing the Swedish mutation (K670N/M671L) (Parvathenani et al., 2003) and rats injected with amyloid-β peptide (Aβ) 1-42 (1 nmol) into the hippocampus (McLarnon et al., 2006) (Figure 3).

Further, the involvement of two P2X7 receptor SNPs were investigated in AD patients and compared to age-matched non-demented elderly, the 1513A > C (rs3751143) and 489C > T (rs208294) (Sanz et al., 2014). This study showed that the presence of the 1513C allele and the absence of the 489C allele (i.e., the presence of both SNPs) decreased the probability of having AD by about four-fold versus the reference subgroup (Sanz et al., 2014). The 1513A > C substitution is associated to the loss of P2X7 receptor function and should confer an “anti-inflammatory” phenotype (Gu et al., 2001). On the other hand, the 489C > T SNP causes a gain of function of this receptor, which may potentiate P2X7 receptor-induced phagocytosis, and subsequent Aβ elimination (Cabrini et al., 2005; Sluyter and Stokes, 2011) (Figure 2). Therefore, such mutations may be neuroprotective against AD development (Sanz et al., 2014).

Several studies support the idea that the prolonged activation of P2X7 receptor may result in increased secretion of pro-inflammatory cytokines (such as IL1-β and IL-18) and reduced phagocytic capacity, leading to neuronal damage (Skaper et al., 2006; Sanz et al., 2009; Lee et al., 2011). In accordance with this proposal, injection of fibrillar amyloid-β peptide (fAβ1-42) into the dentate gyrus of the hippocampus enhanced microglial reactivity, astrogliosis and leakiness of the blood-brain barrier (Ryu and McLarnon, 2008a, b). Interestingly, a pronounced increase of P2X7 receptor immunoreactivity was detected in astrocytes and microglia, but not in neurons (McLarnon et al., 2006; Ryu and McLarnon, 2008a). Aβ1-42 treatment also induced ATP release, [Ca^2+^]_i_ enhancement and IL-1β secretion in primary microglial cell cultures prepared from wild-type, but not from P2X7 receptor KO mice (Sanz et al., 2009). Intra-hippocampal injection of Aβ1-42 caused a large accumulation of IL-1β in wildtype, but not in P2X7 receptor KO mice (Sanz et al., 2009). Treatment with Bz-ATP increased IL-1β secretion from human microglia cells pre-activated with Aβ1–42, which was completely reversed following pre-incubation with oxidized ATP, P2X7 receptor antagonist (Rampe et al., 2004). This response may be mediated by P2X7 receptors, since the treatment with the selective P2X7 receptor antagonist A-740003 blocked the release of IL-1β induced by ATP treatment of microglial cells from rat cortex incubated with serum amyloid A (Figure 3).

Further evidence indicated that P2X7 receptor activation may also induce neuronal damage in AD through the production of reactive oxygen species (ROS). In microglial cultures, Aβ1-42 induced ATP release leading to the production of ROS via P2X7 receptor activation (Soo et al., 2007). A catalytic subunit of NADPH oxidase, which catalyzes the production of ROS, was detected in P2X7 receptor-positive microglial cells in the cerebral cortex of 6-months-old APP/PS1 mice, a double transgenic mice commonly used to study familial AD (Lee et al., 2011). Moreover, postsynaptic density 95-positive dendrites showed significant damage in P2X7 receptor-positive regions in the cerebral cortex of these animals (Lee et al., 2011). Up-regulation of P2X7 receptor expression and ROS production in microglia cells were temporally correlated with Aβ increase and synaptotoxicity in this animal model, since it occurs around the age of 6 months (Lee et al., 2011).

Studies demonstrated that P2X7 receptor activation interferes with processing of APP. APP is proteolytically processed by β- and γ-secretases to release Aβ, the main component of senile plaques found in the brains of AD patients (Zhang et al., 2011). Alternatively, APP can be cleaved by α-secretase, leading to the formation of the nonpathogenic amyloid-α peptide (Aα) (Zhang et al., 2011). In two different cellular lines (HEK293T and neuroblastoma N2a), inhibition of either constitutive expression or overexpression of the P2X7 receptor increased α-secretase activity through inhibition of glycogen synthase kinase 3 (GSK-3) (León-Otegui et al., 2011; Diaz-Hernandez et al., 2012; Miras-Portugal et al., 2015). In addition, systemic administration of P2X7 receptor antagonists in APP_SweInd_ mice, a transgenic animal that expresses the human APP bearing both the Swedish (K670N/M671L) and the Indiana (V717F) mutations, decreased the number of hippocampal amyloid plaques (Diaz-Hernandez et al., 2012; Miras-Portugal et al., 2015). This reduction is correlated with a decrease in GSK-3 activity and consequent increase of α-secretase activity, leading to non-amyloidogenic APP processing (Diaz-Hernandez et al., 2012; Miras-Portugal et al., 2015).

However, results from Delarasse et al. (2011) showed the opposite effect: P2X7 receptor stimulation may enhance α-secretase activity. In this work, four different cell lines (mouse and human neuroblastoma cells, primary murine astrocytes and neural progenitor cells) incubated with ATP or Bz-ATP had activated enzymatic cascades that triggered α-secretase activity, leading to increased levels of Aα, while Aβ was undetectable (Delarasse et al., 2011). Moreover, this study provides evidence to support the idea that ATP- or Bz-ATP-mediated Aα release is mediated by P2X7 receptor activation: (1) three pharmacological inhibitors of P2X7 receptor blocked the release of Aα mediated by Bz-ATP; (2) inhibition of P2X7 receptor synthesis by RNA interference reduced Aα production; and (3) stimulation by Bz-ATP of mouse primary astrocytes and neural progenitor cells from P2X7 receptor-deficient mice did not induce Aα release, while it did in cells derived from wild type animals (Delarasse et al., 2011). Despite such interesting data, it is relevant to emphasize that APP processing depends on the abundance of this protein at the specific cellular model and, in this case, the equilibrium between the different proteolytic pathways could be unbalanced, which could explain the contrast with the results obtained by other authors (León-Otegui et al., 2011; Diaz-Hernandez et al., 2012; Miras-Portugal et al., 2015). Therefore, the roles of P2X7 receptors in α-secretase activity and APP processing are controversial and should be further investigated. In addition to the aforementioned effects mediated by P2X7 receptors, these receptors have also been involved in memory and cognition impairment, key symptoms of AD frequently attributed to Aβ deposits and neurofibrillary tangles, which spread from the trans-entorhinal and hippocampal regions to the primary areas of the neocortex (Raskin et al., 2015). In accordance with the detrimental role of P2X7 receptor activation in AD, systemic administration of a P2X7 receptor antagonist, BBG, diminished spatial memory impairment and cognitive deficits along with reduced loss of filopodia and spine density induced by the injection of soluble Aβ1-42 into the hippocampal CA1 region of mice, an animal model of AD (Chen et al., 2014). BBG also inhibits, at a lesser extent, P2X4 receptors, which could be responsible for the observed neuroprotective effects. Knockdown of the P2X4 receptor attenuated Aβ1-42-induced neuronal death in neurons primary culture, whereas induction of P2X4 receptor expression in a neuronal cell line that does not express P2 receptors enhanced the toxic effect of Aβ1-42 (Varma et al., 2009).

However, other authors observed that P2X7 receptor inhibition may induce memory deficits. For instance, P2X7 receptor KO mice displayed spatial memory impairment in the Y-maze test, despite their performances in the object recognition task remained unaltered (Labrousse et al., 2009). Additionally, P2X7 receptor KO mice or wild type animals treated with A-438079 presented increased contextual fear recall and impaired acquisition of extinction in mice (Domingos et al., 2018). The treatment with A-740003 elicited dose-dependent impairments in memory acquisition, consolidation and retrieval in rats, whereas P2X7 receptor deletion hampered the aversive memory processes of mice exposed to the contextual fear-conditioning task (Campos et al., 2014). The obtained results indicate that P2X7 receptor inhibition induces memory impairment associated to anxiogenic-like responses. At this point, it is important to highlight that such studies were not conducted in an animal model of AD, but in tests used to evaluate memory and anxiety-related behaviors. The opposite effect observed in an animal model of AD is understandable since experimental conditions were different.

Altogether, literature data indicates that P2X7 receptor inhibition: (1) ameliorates neuronal damage induced by both neuroimmune response activation and ROS production; (2) modulates α-secretase activity and non-amyloidogenic APP processing, in a non-elucidated manner; and (3) attenuated spatial memory impairment and cognitive deficits in an animal model of AD. These results support that P2X7 receptor antagonism may be a possible strategy for AD treatment.

### Parkinson’s Disease

Parkinson’s disease is a neurodegenerative disease that affects more than 1% of the world’s elderly population (between 60 and 80 years old) (de Lau and Breteler, 2006). Despite its high incidence, PD etiology is still poorly understood. Dopaminergic neurons of the nigrostriatal pathway undergo neurodegeneration, accompanied by neuroinflammation and oxidative stress. The appearance of protein aggregates formed by α-synuclein aggravating the disease state is also one of the hallmarks of the disease, although it is not the main cause of dopaminergic neuron death (Hornykiewicz, 1966; Hughes et al., 1992; de Lau and Breteler, 2006).

Patients with PD have characteristic symptoms, such as shaking palsy, resting tremor and bradykinesia, as well as non-motor symptoms, including cognitive impairment and mood and sleep disorders (Thenganatt and Jankovic, 2014). Current treatments consisting of remission of symptoms trigger several adverse effects that compromise the quality of life of the individual. There is no known cure for the disease, highlighting the importance of elucidating the mechanisms involved in the disease and possible therapeutic targets (Hornykiewicz, 2002).

In humans, genetic predisposition to PD development was identified in patients carrying P2X7 receptor polymorphisms. In a Han Chinese population, the P2X7 receptor polymorphism rs3751143 (Glu496Ala) was identified as a risk factor for PD (Liu et al., 2013) (Figure 2).

Animal models of PD show that the P2X7 receptor is involved in disease development, especially in microglial cell activation. In an animal model of nigrostriatal injury induction by 6-OH dopamine (6-OHDA), a toxic dopamine analog, striatal gene expression of the P2X7 receptor gradually increased over 5 weeks after injury (Oliveira-Giacomelli et al., 2019). Neuroprotective effects of P2X7 receptor antagonism were observed after pretreating animals with A-438079. This treatment prevented the decrease in striatal dopamine stocks triggered by 6-OHDA injection. However, this effect was not accompanied by a reduction of dopaminergic neuron death, indicating that P2X7 receptor inhibition acts on axonal dopamine stores (Marcellino et al., 2010) (Figure 3).

Similar results were obtained with BBG treatments. When administered prior to induction of the 6-OHDA injury, intracerebroventricular injection of BBG also protected against decreasing striatal dopamine levels and reduced oxidative stress, mitochondrial dysfunction and apoptosis (Kumar et al., 2017). Treatment with BBG (45 mg/kg) in rats prevented the reduction of striatal and nigral dopamine levels, decreased astrogliosis, striatal microgliosis, and the number of apomorphine-induced rotations (Carmo et al., 2014). Controversially, Hracskó et al., 2011 showed that P2X7 receptor KO animals are equally susceptible to dopaminergic neuron death induction by MPTP (Hracskó et al., 2011). In this study, the Pfizer KO mouse strain was used, known to express P2X7 13C and 13B receptors in the brain (Bartlett et al., 2014).

Additionally, it is suggested that P2X7 receptor inhibition may also promote neuroregeneration of dopaminergic neurons when given 1 week after 6-OHDA-induced injury (Ferrazoli et al., 2017; Oliveira-Giacomelli et al., 2019). Administration of BBG (50 mg/kg) in rats during 7 days, starting 1 week after injury, augmented the number of substantia nigra dopaminergic neurons (Ferrazoli et al., 2017). Likewise, BBG (75 mg/kg) treatment also regenerated striatal dopaminergic fibers. This effect was accompanied by decreased microglial activation in the substantia nigra (Oliveira-Giacomelli et al., 2019) (Figure 3).

Treatment of neuronal-differentiated SH-SY5Y cells, an *in vitro* model of dopaminergic neurons, with BBG protected cells from 6-OHDA-induced synaptotoxicity and death (Carmo et al., 2014; Oliveira-Giacomelli et al., 2019). In addition, assays with wild-type and α-synuclein mutants of microglial cells showed that α-synuclein activated microglial P2X7 receptors, inducing NADPH oxidase, modulating the PI3K/AKT signaling pathway and increasing oxidative stress (Jiang et al., 2015). Subsequently, it has been reported that this α-synuclein-promoted effect on microglial cells *in vitro* also involves the stimulation of glutamatergic excitotoxicity (Dos-Santos-Pereira et al., 2018).

Overall, P2X7 receptor inhibition presents neuroprotective and neuroregenerative effects in cellular and animal models of PD. This effect involved anti-inflammatory actions and modulation of the microglial activation state and cytokine release. However, most of these studies used BBG as a tool to assess P2X7 receptor antagonism. Therefore, we cannot discard that P2X4 receptors could be partially responsible for neuroprotective and/or neuroregenerative effects in PD’s models (Ase et al., 2015). P2X4 receptor inhibition did not prevent 6-OHDA-induced cell death in SH-SY5Y cell culture (Oliveira-Giacomelli et al., 2019). This result indicates that P2X4 receptor antagonism is not the main mechanism of neuroprotective effect of BBG treatment. On the other hand, there is no reported study of P2X4 receptor antagonism inducing neuroregenerative effects. Thus, P2X4 receptor antagonism could be partially responsible for the regeneration of dopaminergic neurons in the animal model of PD induced by 6-OHDA. In conclusion, P2X7 receptor is an interesting research topic and possible target for PD.

### Huntington’s Disease

Huntington’s disease (HD) is a dominant hereditary disease caused by a mutation in IT15 gene that encodes huntingtin protein (Htt). Abnormal elongation of the (CAG)n repeats localized in 5′ coding sequence results in massive neurodegeneration of the basal ganglia and cortex of patients over the age of 30 (Vonsattel and DiFiglia, 1998; Ross and Tabrizi, 2011; Ross et al., 2014). The role of P2X7 receptor in HD has been still poorly investigated. At the moment, the only study is published by Diaz and collaborators, who by using two distinct mouse models for HD, Tet/HD94 and R6/1 demonstrated that P2X7 receptor expression is increased in HD, and that the receptor channel possesses augmented Ca^2+^ permeability (Díaz-Hernández et al., 2009) (Figure 3). The inhibition of the receptor with BBG mitigated motor coordination deficits, cachexia and decreases neuronal loss.

Moreover, *in vitro* analysis revealed that neurons expressing mutant Htt were more sensitive to apoptosis under P2X7 receptor stimulation (Díaz-Hernández et al., 2009). Thus, P2X7 receptors expressed in microglia can promote excitotoxicity in neural cells by inducing glutamate release (Matute, 2012).

### Amyotrophic Lateral Sclerosis

Amyotrophic lateral sclerosis (ALS) is one of the most prevalent neuromotor diseases in adulthood. The disease is characterized by the death of motoneurons in the motor cortex, brainstem and spinal cord, resulting in muscle impairment and paralysis (Hardiman et al., 2017). Among the mechanisms involved in neuronal death, neuroinflammation is one of the most established factors. ALS patients present alterations in levels of a range of pro-inflammatory cytokines in the cerebrospinal fluid (Mitchell et al., 2009; Moreno-Martinez et al., 2019), as well as increased rates of reactive cerebral microglial cells (Turner et al., 2004). Depending on the stage of the disease, reactive microglia with protective or cytotoxic properties is found, demonstrating the complexity of neuroinflammation in this disorder (Evans et al., 2013). In this sense, studies relating the P2X7 receptor with ALS show a delicate regulation depending on different factors.

Several studies have been conducted with superoxide dismutase 1 transgenic mice harboring the G92A mutation [SOD1 (G93A)], a well-known ALS model. In this model, onset, progression, and animal survival depend on the mouse gender. Cervetto et al. (2013) showed that inhibition of the P2X7 receptor by BBG at a dose of 45 mg/kg slowed down disease progression in males, but not in females (Cervetto et al., 2013).

In addition, Apolloni et al. (2013a) demonstrated that female SOD1 (G93A) mice with the KO of the P2X7 receptor gene showed increased survival but anticipated the onset of the disease and intensified its progression in males and females. Further, increased astrogliosis and microgliosis and augmented motoneuron death were observed, accompanied by increased pro-inflammatory cytokine production (Apolloni et al., 2013a). Authors used Pfizer KO mice, known to express P2X7 13B and 13C receptors in the brain, which present lower membrane migration and channel function when compared to P2X7A receptors (Masin et al., 2012).

The beneficial effects of P2X7 receptor blockade in ALS supposedly did not depend only on the studied gender, but also on the stage of the disease. In the ALS pre-onset phase in SOD1(G93A) mice, Bartlett et al. (2017) used BBG at a dose of 45 mg/kg, three times a week. They reported that this treatment increased female survival without ameliorating motor performance (Bartlett et al., 2017).

Corroborating these results, treatment of late-pre-onset SOD1 (G93A) mice with BBG at 50 mg/kg, three times a week, delayed disease onset and improved motor performance (Apolloni et al., 2014). In addition, this treatment increased motoneurons survival and decreased microgliosis and expression of pro-inflammatory markers. However, when treated in the onset phase, no neuroprotective effect was observed by P2X7 receptor antagonism. On the other hand, P2X7 receptor activation exerted a protective effect on skeletal muscles of SOD1 (G93A) mice (Fabbrizio et al., 2019). Pre-late-onset treatment with Bz-ATP at a dose of 1 mg/kg for 7 days (i.p.) prevented denervation atrophy of the skeletal muscle. The neuroprotective effect of Bz-ATP could be attributed to another purinergic receptor since this compound is not a selective agonist of P2X7 receptors. Despite that, the P2X7 receptor is known to control proliferation, differentiation, and regeneration in healthy skeletal muscle (Figure 3).

*In vitro*, the co-culture of astrocytes and motoneurons from SOD1 (G93A) mice showed P2X7 receptor involvement in astrocyte activity. The addition of Bz-ATP and ATP induced motoneuron death by astrocytic neurotoxicity. When BBG or apyrase (that increases ATP metabolism and decreases its concentration) was used, inhibition of neuron death was observed (Gandelman et al., 2010). Although BBG treatment also inhibits P2X4 receptors, activation of these receptors appears to protect motor neurons *in vitro* (Cieślak et al., 2019), indicating that the P2X7 receptor subtype is more likely to be activated in the detrimental effect of Bz-ATP. Subsequently, BBG treatment of motoneurons isolated from rat embryonic spinal cord prevented Bz-ATP-induced cell death. In addition, although low concentrations of ATP induced neuronal death, high concentrations of ATP in the cellular media exerted a protective effect, possibly due to its hydrolysis in ADP and the adenosine-induced activation of P1 receptors. ATP and Bz-ATP induced apoptosis by peroxynitrite production, p38 activation and stimulation of the FAS autocrine signaling pathway (Gandelman et al., 2013).

*In vitro* studies also corroborate microglial participation in disease development in SOD1 (G93A) mice. Using isolated microglia from these animals, Apolloni et al. (2013b) demonstrated that Bz-ATP increased ROS production and activation of the ERK1/2 signaling pathway (Figure 3). The pro-inflammatory effects were alleviated following BBG application. Similar results were obtained in SOD1 (G93A) P2X7 receptor KO microglial cells, strengthening the concept of anti-inflammatory effects promoted by P2X7 receptor antagonism (Apolloni et al., 2013a). Besides inducing pro-inflammatory effects, activation of P2X7 receptors in microglia cells isolated from SOD1 (G93A) mice supposedly also modulate autophagy processes. Bz-ATP increased expression of autophagy markers by inhibiting mTOR phosphorylation. This effect was attenuated by treatment with the P2X7 receptor antagonist A-804598 (Fabbrizio et al., 2017).

Finally, peripheral blood mononuclear cells of patients with ALS showed decreased P2X7 receptor expression. Repeated application of ATP to these cells resulted in diminished intracellular calcium transients compared to controls, demonstrating that decreased P2X7 receptor expression induced dysregulation of intracellular calcium homeostasis (Liu et al., 2016).

In conclusion, P2X7 receptor inhibition supposedly promotes dual effects along the course of ALS. Its effects seem to depend on the time window in which the inhibition started. P2X7 receptor ablation before ALS development in mice seems to be detrimental (Apolloni et al., 2013a). In the asymptomatic phase, P2X7 receptor inhibition did not alter disease onset and survival, although it decreases M1 microglial marker expression (Apolloni et al., 2014). In the pre-onset phase, treatment with BBG increased mice’s survival but did not alleviate motor symptoms (Bartlett et al., 2017). When administered at the late pre-onset phase, BBG reduced M1 microglial phenotype and increased anti-inflammatory M2 phenotype along with delayed disease onset and decreased motor symptoms (Apolloni et al., 2014). BBG is known to also inhibit P2X4 receptors to a lesser extent, but the role of P2X4 receptors in the ALS development depends on the cell type. While P2X4 receptor inhibition in microglia cells induces the phenotypic change to M1 microglial cells and promotes inflammation, P2X4 receptor activation appears to protect motor neurons against kainate-induced excitotoxicity *in vitro* (Di Virgilio and Sarti, 2018; Cieślak et al., 2019). Since BBG treatment induced a decrease in microglial M1 markers, it is more likely that the neuroprotective effects of BBG treatment involves P2X7 receptor inhibition rather than P2X4 receptor inhibition in ALS.

### Multiple Sclerosis

Multiple sclerosis is an autoimmune disease with unknown etiology. It is characterized by chronic inflammation with astrogliosis and microgliosis, death of oligodendrocytes, axonal demyelination and subsequent neuronal transmission impairment. Available drugs alleviate symptoms; however, there is no known cure for this disease (Goldenberg, 2012). Sustained activation of the P2X7 receptor is known to induce oligodendrocyte death and demyelination and neuroinflammatory processes and neurodegeneration, which are characteristic for MS. Thus, studies unraveling functions of this receptor in MS development were conducted.

An animal model of autoimmune encephalomyelitis (EAE) is the gold-standard tool for *in vivo* studies, presenting similar features of MS (Lassmann, 1983). In EAE animals, injection of 10 mM BBG into the optic nerve reduced ATP and Bz-ATP-induced demyelination, suggesting that P2X7 receptor activation induced oligodendrocyte excitotoxicity (Matute et al., 2007). BBG also inhibit P2X4 receptors, but their activation in microglia cells is proposed to trigger remyelination process in EAE mice (Di Virgilio and Sarti, 2018), indicating that P2X7 receptor antagonism could be the responsible for BBG treatment protective effects. P2X7 receptor expression during EAE development in rodents has been demonstrated. In the asymptomatic phase of the disease, overexpression of the receptor in astrocytes was observed. At the peak of the characteristic symptoms of the disease, receptor overexpression occurred not only in astrocytes but also in neuronal terminals (Grygorowicz et al., 2010). Following recovery from the disease, the animals showed P2X7 receptor overexpression in glial cells, whose GFAP labeling was increased in the symptomatic phase without reduction after recovery (Grygorowicz et al., 2011) (Figure 3). These results were later confirmed, in which reactive astrocytes in the early phase of the disease expressed P2X7 receptors. Treatment with BBG (50 mg/kg) for 6 days alleviated the appearance of the characteristic symptoms of the EAE rat model, accompanied by reduction in reactive astrocyte labeling (Grygorowicz et al., 2016). Microglial cell analysis also yielded interesting results. In the asymptomatic phase of EAE, microglial cells showed P2X7 expression in active and resting phenotypes, and treatment with 50 mg/kg BBG for 6 days reduced microglial activation and pro-inflammatory cytokine release (Grygorowicz and Strużyńska, 2019).

In the Pfizer P2X7 receptor KO animals, induction of the EAE model resulted in a more severe pathological scenario of the disease. Moreover the authors of this study (Chen and Brosnan, 2006) injected bone marrow cells from P2X7 receptor KO mice into wild-type animals and detected a greater susceptibility to the disease. *In vitro* co-culture of P2X7 receptor KO macrophages and lymphocytes revealed increased lymphocyte proliferation together with decreased apoptotic activity. These results suggest that enhanced disease susceptibility of P2X7 receptor KO animals may be due to decreased lymphocyte apoptosis rates (Chen and Brosnan, 2006). Controversially, Sharp and colleagues showed that GlaxoSmithKline P2X7 receptor KO mice presented four times less development of the EAE model, with reduced astrocyte activation and axonal damage. On the other hand, they detected an increase in pro-inflammatory cytokine production in splenic T-cells (Sharp et al., 2008), explained by expression of P2X7K receptors in these cells (Bartlett et al., 2014). Although controversial, these results ensure that P2X7 receptors play an important role in the development of the EAE model, both peripherally and in the central nervous system.

Activation of P2X7 receptors is known to induce opening of pannexin-1 associated membrane pores, with increased release of ATP. In this sense, pannexin-1 KO mice showed a decrease in EAE onset rates, accompanied by diminished mortality. In addition, ATP release in the spinal cord was diminished, accompanied by an increase in P2X7 receptor expression. In the long term, these animals developed symptoms as severely as wild-type animals did when submitted to the EAE model. The authors of the work (Lutz et al., 2013) suggested that increased P2X7 receptor expression is a mechanism to counteract the decrease in ATP release due to the absence of pannexin-1, and that this mechanism may be the reason for the similar development of symptoms. When treated with the pannexin-1 inhibitor mefloquine wild type EAE animals showed less severity in EAE development (Lutz et al., 2013).

The P2X7 receptor is associated with reactive microglia, as shown for microglial cells extracted during the autopsy of individuals with MS (Beaino et al., 2017). In addition, P2X7 receptor activation may play a role in the upregulation of IL-1β through nitric oxide synthase expression (Narcisse et al., 2005). P2X7 receptor expression was detected in reactive astrocytes in postmortem brains, showing expression upregulation in the parenchyma of the frontal cortex and in microglial cells from spinal cord and white brain matter (Narcisse et al., 2005; Yiangou et al., 2006; Amadio et al., 2017). P2X7 receptor expression was reduced in peripheral blood mononuclear cells (PBMCs) during acute disease phase, possibly due to autocrine and paracrine mechanisms resulting from inflammatory processes. The obtained results indicate that P2X7 receptor expression downregulation in monocytes and upregulation of expression in astrocytes participate at the inflammatory process of MS (Amadio et al., 2017). In contrast, PBMCs from MS patients had no difference in P2X7 receptor expression when compared to healthy individuals (Caragnano et al., 2012). However, when treated with glatiramer acetate, a compound used for MS treatment, P2X7 receptor and CD39 expression rates were reduced in PBMCs. These data were corroborated by *in vitro* studies of PBMCs, which when treated with glatiramer acetate showed a decrease in P2X7 receptor expression and a tendency to reduced IL-1β and increased CD39 expression (Caragnano et al., 2012).

Besides rare mutations in the P2X7 receptor gene found in familial MS (Sadovnick et al., 2017; Zrzavy et al., 2019), patients with mutations of Arg307Gln (rs28360457), which cause a substantial loss in membrane pore formation, are up to twice less frequent in MS patients, indicating a protective effect of this mutation (Gu et al., 2015). The opposite occurs when the mutation involves a P2X7 receptor gain-of-function that increases receptor channel permeability for Ca^2+^ such as the Ala76Val polymorphism, which is more common in MS patients (Oyanguren-Desez et al., 2011) (Figure 2).

Altogether, *in vivo* and *in vitro* evidence in animal models and patient samples indicates that the P2X7 receptor is closely related to MS pathology. Its expression is increased in microglia and reactive astrocytes resulting from inflammatory processes, and interventions that downregulate expression or activity of this receptor have neuroprotective effects. Moreover, although several studies used BBG as antagonist for P2X7 receptors, and this compound also inhibits P2X4 receptors, activation of the latter is known to induce microglial changes towards the M2 phenotype exerting remyelination effects in EAE mice (Di Virgilio and Sarti, 2018). Additionally, outcomes of P2X7 receptor ablation before EAE development are not clear, since different P2X7 receptor KO mice present different outcomes.

## P2X7 Receptor Roles in Psychiatric Disorders

As reviewed by Cheffer et al. (2018), a range of purinergic receptors are involved in psychiatric disorders. As discussed below, the P2X7 receptor also seems to influence development, vulnerability and severity of these disorders.

### Depressive Disorders

Major depressive disorder (MDD) is estimated to affect about 322 million people worldwide, which represents 4.4% of the global population (World Health Organization, 2017). Prevalence rates vary by sex (5.1% of females and 3.6% of males) and by age (peaking in the older adulthood, between 55 and 74 years old) (World Health Organization, 2017). As described by several studies, MDD has a high social and economic impact (Wang et al., 2003; Greenberg et al., 2015), which could be attenuated by more appropriated treatments (Chisholm et al., 2016). However, about 65% of patients with MDD fail to achieve remission and about 33% do not respond to the treatment initially prescribed (Schatzberg, 1999; Trivedi et al., 2008). A possible explanation for the ineffectiveness of antidepressants in some patients is that most of them acts through facilitation of monoaminergic neurotransmission and studies from the last decade show that depression etiology involves more than this system (Kendler et al., 2006; Dean and Keshavan, 2017).

Depressive disorders result from a combination of environmental influence, personality traits, genetic and epigenetic factors leading to neuroendocrine dysfunction (hypothalamic–pituitary–adrenal axis imbalance), neurochemical alterations (impaired monoaminergic neurotransmission, increased glutamate levels and enhanced neuroimmune response) and decreased neuroplasticity (Kendler et al., 2006; Dean and Keshavan, 2017). As recently reviewed by Ribeiro and co-workers the P2X7 receptor is a core regulator of such neurochemical and neuroplastic mechanisms (Ribeiro et al., 2019a). Based on that, it is not surprising that several studies indicate P2X7 receptor involvement in mood disorders as discussed in the following.

A pioneering work showed an association between the presence of the SNP rs2230912 in the gene coding for P2X7 receptor with MDD development (Lucae et al., 2006). Accordingly, the SNP rs2230912 was also associated with mood disorders, longer depressive episodes (Soronen et al., 2011) and increased severity of the depressive symptoms (Hejjas et al., 2009). However, the case-control study performed by Hejjas et al. (2009) found no differences in the presence of these polymorphisms between patients suffering from MDD and controls. Moreover, opposite results were found by two meta-analysis studies: Feng et al. (2014) reported that there was no association between rs2230912 polymorphism and MDD development; however, Czamara et al. (2018) showed a positive correlation (Feng et al., 2014; Czamara et al., 2018) (Figure 2). It is noteworthy that the latter work included more validated studies, which could explain the different results.

In addition, mice expressing either normal human P2X7 receptors (hP2X7 receptor – wild type) or receptors expressed by an altered gene (hP2X7 receptor – rs2230912), did not present any behavioral changes (Metzger et al., 2017b). However, hP2X7 receptor – rs2230912 mice showed increased vulnerability to chronic social defeat stress. These results indicate that heterozygotic individuals may be more susceptible to development of MDD through interactions between genetic predisposition and stress exposure (Metzger et al., 2017b). In accordance with this idea, the gene polymorphism rs7958311 in P2X7 receptor was correlated with MDD development in individuals with previous history of stress exposure (Gonda et al., 2018) (Figure 2).

Beyond the evidence provided by human studies, *in vitro* and *in vivo* experiments may also help to understand the role of the P2X7 receptor in depression and in the mechanisms underlying therapeutic and/or side effects induced by antidepressants. For this purpose, the effects of antidepressant treatment on the expression/function of the P2X7 receptor has been investigated. In a whole-cell patch-clamp study, paroxetine, but not fluoxetine nor desipramine administration, reduced the inward currents evoked by Bz-ATP on cloned rat P2X7 receptors expressed in HEK293 cells (Wang et al., 2016). In another study, paroxetine inhibited, while fluoxetine and clomipramine potentiated ATP-induced dye uptake in HEK-293 cells expressing recombinant human P2X7 receptors (Dao-Ung et al., 2015). *In vivo*, antidepressant-like effect induced by clemasine (Su et al., 2018), ketamine (Tan et al., 2017) and imipramine (Ribeiro et al., 2019b) were associated with diminished P2X7 receptor levels in the hippocampus of stressed animals. These results suggest that P2X7 receptor activity/expression can be modulated by different antidepressants, revealing a potential mechanism by which these drugs may induce their therapeutic effects. Accordingly, mice exposed to chronic unpredictable mild stress (CUMS) (Su et al., 2018) or chronic restraint stress (Tan et al., 2017) presented enhanced P2X7 receptor expression in the hippocampus. However, there are also animal studies showing no alterations (Yue et al., 2017) or even a reduction (Kongsui et al., 2014) in hippocampal P2X7 receptor levels induced by stress exposure. The discrepant data may be explained by different techniques used to determine P2X7 receptor levels (Western blotting versus immunohistochemistry), different stress protocols, or it may indicate a more complex role of P2X7 receptor in stress induced consequences (Figure 3).

Aiming to better understand P2X7 receptor involvement in stress response, the effects of P2X7 receptor inhibition has been studied. P2X7 receptor KO mice presented antidepressant-related behavior in both forced swim test (FST) and tail suspension test (TST), two experimental approaches to predict antidepressant effects of drugs (Basso et al., 2009; Csölle et al., 2013a, b). In addition, P2X7 receptor KO mice demonstrated improved responses to a sub-effective dose of imipramine in the FST (Basso et al., 2009). Despite these results, Boucher and co-workers observed a decrease in the immobility time of P2X7 receptor KO mice only after repeated exposure to the FST (Boucher et al., 2011). Altogether, data from P2X7 receptor KO mice indicate that P2X7 receptor absence results in increased resilience to stress, and a phenotype showing antidepressant-related behaviors.

Pharmacological studies in rodents using antagonists with different affinities for P2X7 receptor further support this hypothesis. Pereira and co-workers observed that acute treatment with PPADS (12.5 mg/kg), a pan antagonist for P2 receptors, or iso-PPADS (12.5 or 25 mg/kg), an antagonist of P2X receptors, decreased the immobility time in the FST (Pereira et al., 2013). Csölle et al. (2013b) observed that systemic administration of BBG at dose of 50 mg/kg/day during 4 days, increased sucrose consumption and decreased the immobility time in the TST of mice pretreated with LPS. In another study from the same research group subchronic (7 days) but not acute treatment with BBG (50 mg/kg/day) decreased the immobility time of mice exposed to TST (Csölle et al., 2013a). Mice systemically treated with BBG (50 mg/kg/day) during 8 weeks (Farooq et al., 2018) or rats treated with A-804598, at a dose of 5 mg/kg twice daily for 4 weeks (Iwata et al., 2016), reversed behavioral alterations induced by CUMS exposure. In accordance with these data, 7 days of treatment with BBG (50 mg/kg/day) decreased the number of escape failures induced by inescapable foot shocks application (Ribeiro et al., 2019b). Additionally, 7 days of treatment with A-804598 (30 mg/kg/day) induced antidepressant-like effects in the flinders sensitive line rats, an animal model of depression based on selective breeding (Ribeiro et al., 2019c). Intracerebral administration of P2X7 receptor antagonists have been also carried out in order to investigate the role of these receptors in specific brain regions. Interestingly, microinjection of P2X7 receptor antagonists (BBG or A-438079) into the rat hippocampus during 3 weeks prevented the development of depression-related behaviors induced by CUMS exposure, while the administration of P2 receptors agonists (ATP or Bz-ATP) for the same period caused depressive-like behaviors similar to those observed after stress exposure (Yue et al., 2017).

Altogether, pharmacological and genetic findings indicate that P2X7 receptor inhibition induces antidepressant-related effects in animals. This response may be mainly associated with the blockade of P2X7 receptors expressed in the hippocampus, although the involvement of other brain structures needs to be further investigated. Regardless the region responsible for the effects induced by systemic administration of P2X7 receptor antagonists, the behavioral response points this receptor as a possible target for depression therapy.

### Bipolar Disorder

Bipolar disorder is an incapacitating, chronic and severe mental disorder that occurs in a cyclic course. Patients with bipolar I disorder (BDI) present an exacerbated mood elevation, mania episodes and usually experience major depression. Bipolar II patients (BDII) exhibit an elevation of mood, named hypomania, and a history of major depression without mania episodes. The whole spectrum of BD is prevalent in approximately 2.4% of population, whereas the prevalence of BDI and BDII are 0.6 and 0.4%, respectively (Merikangas et al., 2011). There is several evidence that BD may progress and present neurodegenerative components, once patients exhibit symptoms worsening, gradual cognitive impairment and brain atrophy (Rao et al., 2010).

The neurobiological processes of BD remain poorly understood. The pathways most associated hitherto include monoaminergic neurotransmission, such as dopaminergic, serotonergic, and noradrenergic systems (Grande et al., 2016), redox imbalance (Versace et al., 2014) and neuroinflammation. Some contradictory results exist regarding the neuroinflammation state in BD. BD is a highly heterogeneous disorder and the classification, cycling phase, number of episodes, and medication can vary widely among patients, which can implicate different inflammatory cytokine patterns present in BD patients. Using a meta-analytic approach, serum or plasma samples evidenced highly concentrated soluble IL-2 receptor, TNF-a, soluble TNF receptor type 1, soluble IL-6, and IL-4 in bipolar patients. Overall, there were not any differences between other analyzed anti-inflammatory and pro-inflammatory cytokines (Munkholm et al., 2013).

Bipolar disorder is extremely difficult to model in rodents since the mechanism behind the maniac and depressive cycle is not well established. Thus, animal models are employed that mimic the state of mania. A mouse strain that naturally presents a mania-like phenotype showed downregulation of P2X7 receptor expression (Saul et al., 2012). In contrast, genetic deletion of P2X7 receptor protected the abnormal locomotor activity by acute amphetamine administration (Csölle et al., 2013b; Gubert et al., 2016). In the mania animal model induced by chronic administration of amphetamine, pharmacological antagonism with A-438079 and genetic deletion of P2X7 receptor completely reverted increased locomotor activity induced by amphetamine (Gubert et al., 2016). Additionally, A-438079 abolished the release of pro-inflammatory cytokines IL-1β and TNF-α and lipid peroxidation in hippocampus (Gubert et al., 2016). Using the same animal model, BBG treatment prevented hyperlocomotion, DOPAC augmentation in the hippocampus, increased NTPDase3 expression and astrogliosis induced by amphetamine (Gubert et al., 2019b) (Figure 3). Although in the last work only the non-specific antagonist BBG was used, Gubert et al. (2016) found similar results when BBG or the specific antagonist A-438079 were administrated. These studies evidence a reproducibility in P2X7 receptor antagonism in the mania model induced by amphetamine, strengthening the possible role of P2X7 receptor in mania-like state in BD.

There are several studies of genetic associations between P2X7 receptor polymorphisms and BD development. However, inconsistent findings made the identification of any association impossible. The rs2230912 is a SNP in the P2X7 receptor gene that promotes gain of function and was previously associated with increased risk of BD development in patients from the United Kingdom and Ireland (McQuillin et al., 2009) and Canada (Barden et al., 2006). Further, BD patients that presented rs2230912 and rs208294 polymorphisms spent more time in the symptomatic stage than patients without these alleles (Soronen et al., 2011). Nevertheless, this finding was not appropriately replicated in other populations studies. A multi-centric analysis conducted in individuals from Germany, Poland, Romania, and Russia evidenced no allelic or genotypic association between rs2230912 and BDI (Grigoroiu-Serbanescu et al., 2009). Studies in Swedish BD patients revealed an association between rs1718119 and rs1621388 polymorphisms and cognitive features of mania – distractibility, thought disorder, and talkativeness. Still, the rs2230912 polymorphism presented no association with BD (Backlund et al., 2011). A study that analyzed nine variants of P2X7 receptor polymorphisms, such as rs591874, rs208293, rs1186055, rs208298, rs503720, rs1718133, rs1718119, rs2230912, and rs1621388, in United Kingdom individuals found that these polymorphisms did not have any effects on BDI susceptibility (Green et al., 2009). A recent study conducted in Brazilian patients evidenced a decrease in 1513C allele frequency and a potential increase in 1513A A/AC genotype frequency of rs3751143 polymorphism in BD patients (Gubert et al., 2019a) (Figure 2). All these polymorphisms in the P2X7 receptor gene represent a gain of function, which could indicate potential influence of the P2X7 receptor behind the genetic predisposal of BD development.

### Schizophrenia

Schizophrenia (SCZ) is a complex, multifactorial, heterogeneous, and severe psychiatric disorder. SCZ symptomatology is classified by three major categories: (1) positive symptoms, in which the patient may present disturbance of thinking, delusions and hallucinations, named psychotic symptoms; (2) negative symptoms that are characterized by impaired motivation, decrease in spontaneous speech, and social withdrawal; and (3) cognitive symptoms, which the core features may present impairments in working memory, attention, problem-solving, and executive functioning (van Os and Kapur, 2009). Many efforts have been placed to understand the molecular mechanisms that cause SCZ, however, the full complexity of this disorder remains unknown. SCZ is a highly polygenic (Owen et al., 2016) and many environmental factors have been already associated (Byrne et al., 2004; Allardyce and Boydell, 2006; Varese et al., 2012; Cantor-Graae and Pedersen, 2013; Moustafa et al., 2017). Besides, it is already known that SCZ is a neurodevelopmental disorder and maternal complications may be risk factors (Khashan et al., 2008; Brown, 2011, 2012; Khandaker et al., 2013). There are multiple lines of evidence supporting the impaired function in dopamine, glutamate and GABA neurotransmission (Schwartz et al., 2012). Similarly, several neurochemical dysfunctions are stated in the kynurenine pathway (Kindler et al., 2019), redox dysregulation (Do et al., 2015), and neuroinflammation (Na et al., 2014; Marques et al., 2019).

Few clinical data are available regarding P2X7 receptor participation in SCZ pathophysiology. Two antipsychotics drugs, prochlorperazine and trifluoperazine, may inhibit human P2X7 receptor function (Hempel et al., 2013). Further, prochlorperazine, a drug with strong antipsychotic action, could act as a negative allosteric modulator of P2X7 receptor activity (Hempel et al., 2013). A populational study conducted with SCZ patients from Denmark analyzed nine SNPs of the P2X7 receptor – rs28360447, rs208294, rs28360457, rs1718119, rs2230911, rs2230912, rs3751143, rs1653624, and rs35933842 – and did not observe any associations between SCZ and these polymorphisms of P2X7 receptor (Hansen et al., 2008) (Figure 2).

It is a tremendous challenge to mimic SCZ using animal models due to its high complexity, multifactorial component, and difficulty to distinguish and analyze positive symptoms of these disorders. Phencyclidine (PCP) is a compound largely used as an inductor for animal models of SCZ once the rodents present some similar features in their behavior. In the acute PCP mouse model, the pharmacological blockade with JNJ-47965567 and genetic deletion of the P2X7 receptor alleviated some behavioral parameters and also alteration of gene expression of GABA receptor subunits and neuregulin 1 in the prefrontal cortex (Koványi et al., 2016). Overall, there is lack of evidence supporting the role of the P2X7 receptor in the neurobiology of SCZ. It is a poorly explored field and more studies are needed to indicate whether or not there is association.

### Anxiety

Anxiety disorders belong to the most prevalent and disabling psychiatric disorders, substantially impacting life quality. It is estimated that 25% of the population will suffer at least one episode of this disease in adulthood. Types of anxiety disorders include separation anxiety disorder, specific phobia, social anxiety disorder, panic disorder, agoraphobia, generalized anxiety disorder, and drug-induced anxiety disorder. Symptoms include anxiety, excessive fear and other mood disturbances (Kessler et al., 2005). Anxiety disorders are often accompanied by other psychiatric disorders, such as MDD and BD (Schaffer et al., 2012).

Current treatments include serotonin and norepinephrine reuptake inhibitors, benzodiazepines and antidepressant drugs. However, these are partially efficient according to patient histories and the type of anxiety disorder (Murrough et al., 2015). Thus, the identification of specific targets for novel therapeutic approaches is urgent.

In PBMCs from patients with anxiety and depression, an increase in P2X7 receptor expression was found after ATP stimulation. In the same cells, patients with comorbidity of anxiety and Sjogren’s syndrome have higher P2X7 receptor expression when compared to control healthy individuals (Xie et al., 2014).

Several studies show the relationship between the chromosome 12q2431, in which the P2X7 receptor gene is inserted, and the development of mood disorders. Thus, polymorphisms of this receptor are widely studied in mood disorders. The SNP rs1718119 with the Thr348Ala mutation was not related to anxiety onset in patients (Erhardt et al., 2007). Although the P2X7 receptor rs2230912 Gln460Arg polymorphism did not present any relation to mood disorders in case-control analysis, this receptor induces higher symptomatic severity scale scores of patients with G-allele (Nagy et al., 2008; Hejjas et al., 2009). In a cohort study, this same SNP was associated with a higher risk of developing mood disorders and alcoholism, including anxiety (Soronen et al., 2011). This study also identified the rs208294 His155Tyr polymorphism as a possible risk factor for disease development (Soronen et al., 2011). In addition, the P2X7 receptor variant rs208294 has been associated with neuroticism-mediated outcomes of mood disorder, a personality trait that indicates vulnerability to the onset of anxiety in stressful situations (Mantere et al., 2012) (Figure 2).

P2X7 receptor KO mice show controversial results regarding anxiety-like behavior. Despite showing decreased depressive behavior, Pfizer P2X7 receptor KO animals showed no anxiolytic effect in the elevated plus maze test (Basso et al., 2009). In contrast, Boucher et al. (2011) found anxiety-like behavior in this same test, but not in the light dark emergence test (Boucher et al., 2011). P2X7 receptor KO mice also exhibited anxiety-like behavior in the elevated plus maze test when subjected to contextual fear condition (Domingos et al., 2018).

The P2X7 receptor also presented discrepant results regarding its involvement in inducing anxiety-like behavior in different animal models. Inhibition of the P2X7 receptor with A-438079 (10 mg/kg) augmented anxiety-like behavior of mice subjected to the contextual fear condition model (Domingos et al., 2018). Antagonism using intraperitoneal injections of A804598 for 25 days decreased this behavior in mice subjected to high fat diet (Dutheil et al., 2016), possibly by blocking the formation of inflammasomes. However, this same compound had also an anxiolytic effect in an unpredictable chronic stress model, blocking the release of IL-1β, TNF-α and inflammasome formation (Iwata et al., 2016).

Overall, effects of P2X7 receptor activity modulation on animal anxiety parameters has yet to be elucidated. Ablation of P2X7 receptor expression did not prevent the onset of symptoms, and receptor antagonism induce pro- and anti-anxiety effects in different animal models.

## Brain Tumors

Brain tumors are intracranial neoplasms that account for 2% of all cancers (Gould, 2018), while being the second most common cancer among 0 to 14-year-old children. Surpassing even leukemia, brain cancers are the leading cause of oncologic death in this age group (American Brain Tumor Association, 2019). Importantly, the brain is a very fertile soil for metastatic seeding, so that brain metastases incidence is estimated to be at least 10 times higher than that of primary brain tumors (Vargo, 2017). In fact, 30% of all people with cancers in other body parts will present brain metastases (Gould, 2018). Among primary malignant brain tumors, 80% of all cases are gliomas, malignant tumors raising from glial cells (Gould, 2018).

Although prognosis greatly varies, the incidence of near- and long-term disabilities is notably high (Mukand et al., 2001). Both the tumor itself and the frequently associated perilesional edema, which can reach a several-fold greater volume than the tumor itself, account for the functional neurological consequences (Tran et al., 2019). Indeed, brain tumors cause severe economic impacts not only due to direct treatment and rehabilitation costs, but also due to productivity loss (Su and Abdullah, 2016).

Among candidate molecular targets for anti-cancer drug development, the P2X7 receptor has received great attention. In fact, high ATP levels are a common feature in the tumor microenvironment, reaching concentrations of up to hundreds of micromolar (Pellegatti et al., 2008), a range of concentration capable of activating P2X7 receptors (North and Barnard, 1997). Thus, it is not surprising that P2X7 receptors emerge as central players of purinergic signaling in the tumor microenvironment. In agreement, P2X7 receptor expression is upregulated in several tumor types (Adinolfi et al., 2002; Slater et al., 2004; Solini et al., 2008; Ryu et al., 2011; Arnaud-Sampaio et al., 2019). Glioma cell lines of human (U-138MG, U-251MG, M059J) (Gehring et al., 2012), rat (C6) (Wei et al., 2008), and mouse (GL261) (Tamajusuku et al., 2010) origin express P2X7 receptors as well. Importantly, glioma cells have decreased sensitivity to the cytotoxic effects of extracellular ATP in comparison to healthy tissue cells (Morrone et al., 2005), and glioma cells show less ATP hydrolysis (Wink et al., 2003), favoring the maintenance of high extracellular ATP concentrations. Furthermore, stimulation by extracellular ATP drives the release of glutamate by GL261 glioma cells, an effect partially reversed by P2X7 receptor antagonism (Strong et al., 2018). Elevated levels of both ATP and glutamate mediate cytotoxic effects on the boundaries of the tumor, favoring its expansion and growth (de Groot and Sontheimer, 2011; Strong et al., 2018).

Brain tumor microenvironment is composed by tumor and stromal cells, as reactive astrocytes, fibroblasts and myeloid-derived cells, including microglia (Volak et al., 2018). Therefore, P2X7 receptor expression in the tumor mass may occur in different cell types, leading to particular downstream responses, which may be pro- or anti-tumoral depending on the context. The analysis of human glioma samples revealed that microglial cells confined within the tumor had increased P2X7 receptor expression, and pharmacological inhibition of the receptor significantly decreased the number of glioma cells (Monif et al., 2014).

In a brain tumor model established by intrastriatal injection of C6 glioma cells in rats, pharmacological inhibition of P2X7 receptor by BBG decreased tumor growth. *In vitro*, BBG treatment decreased the receptor expression and prevented chemotaxis induced by Bz-ATP (Ryu et al., 2011), pointing to a pro-tumoral intrinsic activity of P2X7 receptor in this model. In agreement, stimulation of human glioma cells with Bz-ATP increased cell proliferation and migration, an effect counteracted by an inhibitor of the MEK/ERK pathway, implicating this pathway in P2X7 receptor-mediated proliferative effects (Ji et al., 2018) (Figure 3). Consistently, overexpression of the P2X7 receptor in a naturally low-expressing human glioma cell line conferred modest *in vitro* growth advantages, but largely accelerated tumor growth *in vivo* (Bergamin et al., 2019), reinforcing a trophic role for this receptor. Also, in a mouse model of neuroblastoma, a rare intracranial tumor that affects immature or developing cells of the nervous system, chronic blockade of the P2X7 receptor in tumor-bearing mice diminished progression and metastasis (Ulrich et al., 2018).

In contrast, another study found that P2X7 receptor blockade by BBG increased C6 glioma cell proliferation, an effect corroborated by enhanced tumor growth observed in rats that received intracranial transplantation of C6 glioma cells either due to *p2rx7* gene knockdown or pharmacological P2X7 receptor blockade (Fang et al., 2013). Conflicting findings were attributed by the authors to different periods and doses of BBG treatment, which would lead to distinct microglial responses.

When expressed both in glioma cells and in glioma-infiltrating microglia, the P2X7 receptor mediates the release of pro-inflammatory factors, as monocyte inflammatory protein 1α (MIP-1α) (Fang et al., 2011), monocyte chemoattractant protein 1 (MCP-1) (Wei et al., 2008; Fang et al., 2011; Braganhol et al., 2015), IL-8 (Wei et al., 2008; Braganhol et al., 2015) and VEGF (Wei et al., 2008). In fact, P2X7 receptor expression in tumor bearing-hosts is essential for mounting an effective anti-tumoral immune response, so that genetic deletion or pharmacological blockade of the receptor increased the incidence of tumors in a murine colitis-associated cancer model (Hofman et al., 2015). Furthermore, P2X7 receptor-deficient tumor-bearing mice undergo a shift toward an immunosuppressive response (De Marchi et al., 2019) and show accelerated tumor progression (Adinolfi et al., 2015).

A comparison between human glioma cell lines showed that those with upregulated P2X7 receptor expression exhibited higher sensitivity to irradiation (Gehring et al., 2012). Further studies corroborated that the P2X7 receptor acts synergistically with radiotherapy promoting cytotoxicity, and the level of P2X7 receptor expression is a good prognosis predictor for radiotherapy response in gliomas (Gehring et al., 2015). Treatments with high ATP and Bz-ATP concentrations also potentialized *in vitro* cytotoxic effects of temozolomide, a drug of choice for glioblastoma treatment, in human glioblastoma cells (D’Alimonte et al., 2015). In agreement, the P2X7 receptor is implicated in the ATP-induced necrotic death of glioblastoma murine cells, supporting its role in killing tumoral cells (Tamajusuku et al., 2010), despite the evidence of glioma resistance to ATP-induced cytotoxicity (Morrone et al., 2005).

In summary, responses triggered by P2X7 receptor highly depend on the expression levels of the receptor, on the stimulation tonus and on the cell type, and the context of tumor microenvironment seems crucial for determining whether P2X7 receptor activation will end up being pro- or anti-tumorigenic. Ultimately, translating existing evidence into therapeutically useful approaches demands a fine resolution between the distinct phenomena mediated by P2X7 receptors. Adopting optimized experimental designs is crucial to move forward, highlighting how and when P2X7 receptor actions are relevant for tumoral pathophysiology. Experimental design should take into account the complexity of the tumor microenvironment, the different stages of tumor development and the numerous existing splicing variants of the P2X7 receptor gene. Furthermore, findings should combine multiple strategies and rely on both gene expression modulation tools and specific agonists and antagonists, so that conclusions are reproducible and robust. In fact, a considerable part of the available evidence relies on pharmacological modulators that could target other purinergic receptors, as previously mentioned. IC_50_ values for inhibition of other purinergic receptors by BBG fall in the micromolar range, and experimental concentrations for P2X7 receptor inhibition are traditionally limited to hundreds of nanomolar. However, especially in human cells, in which IC_50_ values for P2X7 receptor and P2X4 receptor inhibition differ by only approximately an order of magnitude, much closer than those observed, i.e., in rats (Jiang et al., 2000), overlapping inhibition of both receptors may occur. In spite of that, evidence implicating P2X4 receptor functions in tumor biology is scarce, and mostly related to its inflammatory roles (Guo et al., 2004). In fact, gliomas poorly express P2X4 receptors, and its presence has no prognostic value (The Human Protein Atlas, 2020).

## Brain-Penetrant P2X7 Receptor Antagonists

As discussed so far, P2X7 receptor blockade may be a viable approach for treating brain diseases. Although a range of P2X7 receptor antagonists were developed, some of them are not capable of passing the blood-brain barrier (Table 1).

Compounds produced by GlaxoSmithKline (GSK-1482160) and Janssen (JNJ-54175446 and JNJ-55308942) were the first to present both effects in rodents and CNS permeability (Letavic et al., 2017; Territo et al., 2017; Chrovian et al., 2018). The observed *in vivo* activity stimulated the use of target engagement assays to drive development of new drugs, as well as allowed pharmacological tests in rodent models of diseases (Bhattacharya, 2018). In this way, GSK and Janssen advanced in developing other P2X7 receptor antagonists capable of penetrating the blood-brain barrier: GSK compound 16 (Beswick et al., 2010), JNJ-42253432 (Letavic et al., 2013; Lord et al., 2014), JNJ-47965567 (Bhattacharya et al., 2013; Letavic et al., 2013), and JNJ-54166060 (Swanson et al., 2016). In addition, Abbott Laboratories synthetized brain-penetrant P2X7 receptor antagonists, namely: A-438079 (Nelson et al., 2006), A-740003 (Honore et al., 2006), A-804598 (Donnelly-Roberts et al., 2009; Able et al., 2011), and A-839977 (Honore et al., 2009).

Despite the development of several compounds [for detailed reviews see Rech et al. (2016), Pevarello et al. (2017)], the only CNS-permeable P2X7 receptor antagonist that advanced to clinical trials was GSK-1482160. Besides promising initial data, the GSK-1482160 did not present the safety margins to achieve such sustained inhibition, and consequently its development was terminated (Ali et al., 2013).

Currently, Affectis Pharmaceuticals disclosed the use of the brain-penetrant P2X7 antagonist AFC-5128 for neuropathic pain and MS treatment, as stated at the company’s website^1^. Moreover, Alzheimer’s Drug Discovery Foundation has been supporting Axxam to identify selective P2X7 receptor antagonists for AD treatment.

## Concluding Remarks

The P2X7 receptor has become a very popular target in the purinergic signaling research. This review collected evidence for P2X7 receptor role in CNS diseases, although further studies are needed for a better understanding of this involvement. The neuroinflammation process is largely prominent in CNS diseases, mainly those covered in this review. It is robustly established that P2X7 receptor activation promotes proinflammatory cytokines release, whereas P2X7 receptor blockade efficiently inhibit the neuroinflammatory process. Additionally, blockade of P2X7 receptor signaling may reduce hippocampal amyloid plaques in AD; regenerate dopaminergic neurons of nigrostriatal pathway in PD; delay the ALS onset, progression, and motor performance; decrease MS-related symptoms and microglial activation in this condition; exhibit anti-depressant properties; reduce features related to mania; and decrease tumor growth. Degeneration of neural cells as presented in these conditions may increase the extracellular ATP levels, leading to overactivation of P2X7 receptors. Furthermore, AD, PD, MS, MDD, and brain tumors present increased P2X7 receptor expression. In view of that, we propose a signal amplification of P2X7 receptors in these diseases.

Pharmacological and genetic studies also contributed to elucidate the neurobiology of these conditions. However, here we provide evidence of the lack specificity of some antagonists and antibodies related to the P2X7 receptor. BBG, for example, is still widely used in the literature due to the low cost and blood-brain barrier permeability despite its non-specificity. Therefore, critical analysis regarding P2X7 receptor studies is extremely necessary.

The most studied SNPs of the P2X7 receptor result in loss or gain-of-function, and several studies associate these SNPs with disease development, symptomatology or disease worsening concerning AD, BD, MS, MDD, PD, and anxiety. Regarding SCZ and anxiety, the role of P2X7 receptor should be further explored to clarify its involvement in the pathogenesis of these disorders. Altogether, the studies presented here show the involvement of the P2X7 receptor in pathologies and the therapeutic potential of inhibiting this receptor in the treatment of brain diseases. Herewith, we suggest that these effects are due to the resolution of neuroinflammation components of the aforementioned diseases.

## Author Contributions

RA wrote the Schizophrenia, Bipolar Disorder, and Conclusion sections and prepared the Figures 2, 3 and Table 1. ÁO-G wrote the Parkinson’s Disease, Amyotrophic Lateral Sclerosis, Multiple Sclerosis, Anxiety, and Conclusion sections and prepared the Table 1. DR wrote the Alzheimer’s Disease, Depressive Disorders, and Brain Penetrant Drugs sections and prepared the Table 1. TG wrote the Introduction and Huntington’s Disease sections and prepared the Figure 1. VA-S wrote the Brain Tumor section. HU and CL conceptualized, supervised manuscript elaboration, edited, revised, and critically overviewed the manuscript. All authors contributed to the article and approved the submitted version.

## Conflict of Interest

The authors declare that the research was conducted in the absence of any commercial or financial relationships that could be construed as a potential conflict of interest.

## Footnotes

1 http://www.affectis.com/afc5128.html
